# Role of exosome therapy in reconstructive surgery: a review

**DOI:** 10.3389/fmed.2026.1697228

**Published:** 2026-03-18

**Authors:** Amr Youssef Arkoubi

**Affiliations:** Department of Anesthesia and Surgery, College of Medicine, Imam Mohammad Ibn Saud Islamic University (IMSIU), Riyadh, Saudi Arabia

**Keywords:** exosomes, extracellular vesicles, reconstructive surgery, regulatory framework, standardization, wound healing

## Abstract

**Introduction:**

Exosomes are nanosized extracellular vesicles that facilitate intercellular communication through their diverse molecular cargo, including proteins, microRNAs, and lipids. By modulating inflammation, angiogenesis, and extracellular matrix remodeling, exosomes have emerged as promising therapeutic agents in plastic and reconstructive surgery. Understanding source-specific characteristics, isolation methods, and delivery strategies is crucial to optimize their clinical translation.

**Methods:**

This review employed a review approach, integrating preclinical and clinical evidence to provide a comprehensive overview of exosome biology, sources, isolation techniques, delivery modalities, and therapeutic applications. Emphasis was placed on highlighting mechanistic insights, translational relevance, and comparative analyses of different exosome sources and delivery strategies. The methodology prioritized thematic organization and qualitative integration over quantitative meta-analysis to capture the breadth of current knowledge and emerging trends in regenerative and reconstructive surgery.

**Results:**

Adipose-derived stem cell (ADSC) exosomes provided high yields with strong angiogenic and anti-fibrotic effects, whereas bone marrow MSC (BM-MSC) and dermal fibroblast exosomes primarily contributed immunomodulatory and extracellular matrix regulatory functions. Umbilical cord MSC (UC-MSC) exosomes showed high proliferative and angiogenic potential. Delivery methods, including topical, hydrogel-based, and direct injection approaches, influenced therapeutic outcomes. Preclinical and early-phase clinical studies reported improved wound closure, scar modulation, and aesthetic outcomes, though the clinical evidence remains predominantly derived from small pilot studies and exploratory trials with limited sample sizes and follow-up durations. Variability in isolation protocols, dosing, and characterization further limited standardization.

**Discussion:**

Exosome-based therapy represents a versatile, minimally immunogenic regenerative approach. Standardized source selection, scalable GMP-compliant isolation, and optimized delivery are essential for reproducibility and clinical translation. Adequately powered randomized controlled trials with long-term follow-up are required to confirm efficacy and establish standardized dosing protocols.

## 1 Introduction

Exosomes [30–150 nanometers (nm)] are nanosized extracellular vesicles of endosomal origin that carry proteins, lipids, and nucleic acids, enabling precise intercellular communication in both physiological and pathological contexts (1). Their therapeutic potential has garnered significant scientific attention, with a marked increase in research output in recent years (2). Their inherent capacity to modulate inflammation, stimulate angiogenesis, enhance collagen deposition, and remodel fibrotic tissue makes them profoundly relevant for complex wound healing, meticulous scar modulation, and sophisticated regenerative procedures in plastic and reconstructive surgery (3, 4).

This burgeoning translational momentum is further underscored by the growing number of formal investigations and investments. A search on ClinicalTrials.gov reveals more than 20 registered clinical trials currently investigating exosome-based interventions for wound healing and scar reduction (5). This clinical interest is supported by increasing venture capital investment in exosome biotechnology companies and is paralleled by evolving regulatory frameworks, including pending draft guidance from the United States Food and Drug Administration (FDA) on minimal manipulation criteria for these products (6). The confluence of these factors places the field at a critical inflection point, demanding evidence-based consensus to prevent disjointed and potentially unsafe translation into mainstream surgical practice. The published literature on exosome therapy has expanded rapidly over the past decade, with a substantial increase in annual publication volume. However, this growth has been accompanied by considerable heterogeneity in study design, experimental models, and outcome measures, necessitating careful interpretation of cumulative evidence.

While preclinical studies have powerfully demonstrated significant therapeutic benefits, including accelerated wound closure in diabetic models (7) and reduced fibrotic response in various injury models (8) clinical translation has yielded notably inconsistent results. For example, a randomized controlled trial (RCT) of exosome-enriched fat grafting reported a compelling 42% reduction in scar thickness with durable outcomes at 12 months (9), yet another RCT for burn scar management surprisingly found no significant improvement over conventional dressings (10). These conflicting findings underscore the preliminary nature of the evidence base and highlight the need for larger, more rigorously designed trials. This striking variability fundamentally arises from a persistent problem: the “black box” nature of current exosome preparations. These preparations are often loosely defined merely by their isolation method rather than a rigorously standardized, molecularly-defined composition, leading to highly variable product quality. Methodological inconsistencies are alarmingly prominent, with over 60% of studies lacking standardized isolation protocols, resulting in substantial and unpredictable variations in vesicle potency and efficacy (11). Regulatory oversight also remains notably fragmented; while no exosome therapies are currently approved by the US FDA or European Medicines Agency (EMA), multiple investigational new drug (IND) applications are under active review (12). In stark contrast, markets like South Korea and Japan permit commercial distribution of cosmetic-grade exosome products under significantly less rigorous regulatory frameworks, creating pronounced global disparities in clinical accessibility and safety standards (13).

This article is structured as a comprehensive review because the field of exosome therapy in plastic and reconstructive surgery is rapidly evolving, highly heterogeneous, and currently lacks standardized methodologies, making a systematic review premature. This review moves decisively beyond a simple summary of the extant literature to critically assess these formidable challenges and propose a unified framework for exosome translation, arguing that the path forward necessitates a profound shift from exploratory, descriptive studies to a highly focused, strategic effort. This effort must be firmly built upon three core pillars: standardized manufacturing, biomarker-based dosing, and regulatory harmonization.

The objectives of this review were to: (a) Provide a comprehensive overview of the biological rationale and therapeutic potential of exosomes in plastic and reconstructive surgery. (b) Summarize and critically evaluate preclinical and early clinical evidence, highlighting strengths, limitations, and translational gaps. (c) Discuss methodological, manufacturing, and regulatory challenges that impede consistent clinical application. (d) Propose a clinical roadmap for standardized exosome therapy, emphasizing manufacturing standards, biomarker-based dosing, and regulatory harmonization.

By addressing these objectives, this review seeks to bridge the translational gap and guide the field toward predictable, consistent, and ultimately, evidence-based clinical implementation of exosome therapy in plastic and reconstructive surgery.

## 2 Biological basis of exosome therapy

### 2.1 Exosome biogenesis: formation and release

#### 2.1.1 Exosome biogenesis: formation and release

**FIGURE 1:**
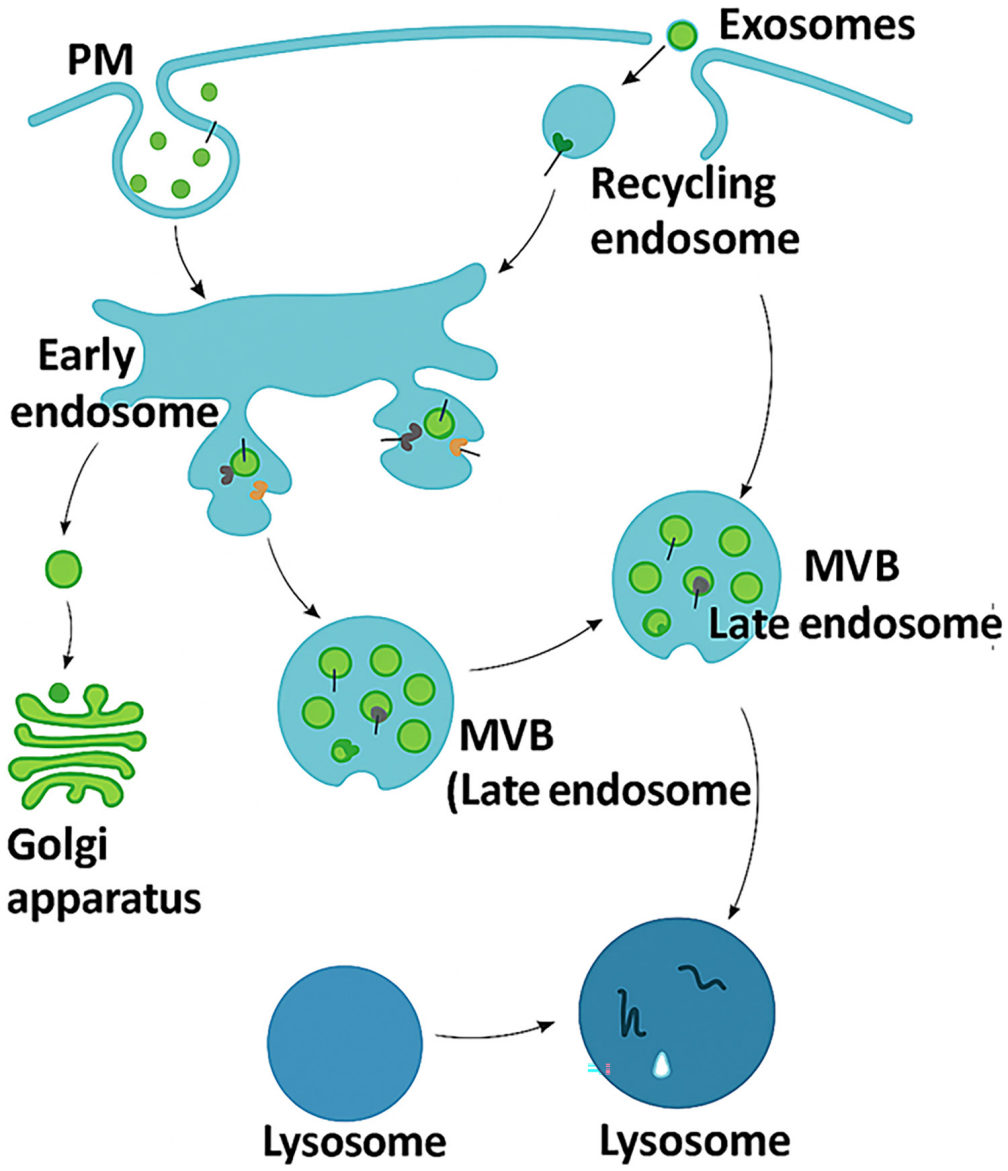
Illustration of exosome biogenesis and key cargo components.

The intricate biogenesis of exosomes begins with the inward budding of the plasma membrane, forming early endosomes. Figure 1 which is a conceptual schematic representation illustrating the dynamic endocytic trafficking pathway, showing how materials internalized from the plasma membrane (PM) are directed to early endosomes for sorting, followed by either recycling to the membrane via recycling endosomes or maturation into late endosomes/multivesicular bodies (MVBs). These MVBs can fuse with the lysosome for degradation or with the plasma membrane to release exosomes, which mediate intercellular communication by transferring proteins and nucleic acids (14). The Golgi apparatus contributes essential components for vesicle formation and sorting, ensuring efficient cargo processing and cellular homeostasis. Disruptions in this endosomal–lysosomal system are associated with pathological processes such as neurodegenerative and metabolic diseases, underscoring the importance of regulated vesicular trafficking in maintaining normal cellular function (15). In exosome-producing cells, this biogenetic cascade is tightly controlled by Rab-GTPases (Rab27a/b, Rab11) and ESCRT complexes, which determine vesicle release efficiency and, ultimately, therapeutic potency. Accurate characterization of these molecular events is crucial for reproducibility in regenerative applications.

This figure represents a conceptual synthesis of established knowledge in extracellular vesicle biology rather than original experimental data.

It depicts the endocytic pathway leading to exosome formation and release. The figure illustrates the inward budding of the plasma membrane to form early endosomes, which mature into late endosomes/multivesicular bodies (MVBs). MVBs either fuse with lysosomes for degradation or with the plasma membrane to release exosomes into the extracellular space. Key molecular components involved in this process include Rab-GTPases (Rab27a/b, Rab11) and ESCRT complexes, which regulate vesicle trafficking and release. The cargo packaged within exosomes comprises tetraspanins (CD9, CD63, CD81), heat shock proteins, nucleic acids (miRNAs, mRNAs), and lipids. This figure represents a conceptual synthesis of established knowledge in extracellular vesicle biology rather than original experimental data.

### 2.2 Molecular cargo and therapeutic function

The molecular cargo encapsulated within exosomes is remarkably diverse and plays a pivotal role in their therapeutic functions. Characteristic exosomal proteins include a family of tetraspanins (CD9, CD63, CD81), which are essential for vesicle formation, precise cargo selection, and efficient recipient cell uptake (16). Other critical proteins, such as heat shock proteins (HSP70, HSP90), specific integrins that aid in tissue targeting, and various enzymes involved in complex cellular signaling are also highly prevalent (17). The nucleic acid cargo primarily comprises microRNAs (miRNAs), which are small non-coding RNAs that potently regulate gene expression, profoundly influencing critical cellular processes such as cell proliferation, apoptosis, and differentiation (18). The exosomal lipid bilayer, a crucial structural component, is composed of cholesterol, sphingomyelin, and ceramides, providing essential membrane stability and containing bioactive lipids that further enhance targeting capabilities, membrane fusion, and cellular internalization processes (19). Cargo composition varies according to both the source cell and isolation technique; for example, ultracentrifugation tends to enrich protein fractions, whereas tangential-flow filtration preserves miRNA content. Furthermore, vesicles produced under good manufacturing practice (GMP) conditions display more consistent particle size distribution and sterility profiles than laboratory-grade preparations, reducing variability in clinical efficacy.

These diverse components collectively enable exosomes to serve as sophisticated mediators of intercellular communication, making them potent therapeutic tools in regenerative medicine. The specific cargo profile varies significantly depending on the parent cell, which in turn dictates the exosome’s unique therapeutic properties. For example, exosomes derived from mesenchymal stromal cells (MSCs) are rich in anti-inflammatory miRNAs and growth factors that are ideal for reducing scarring and promoting wound healing, while those from platelets contain factors that accelerate hemostasis and epithelialization. Table 1 provides a comparative summary of how the distinct cargo of exosomes derived from MSCs, fibroblasts, and platelets translates to their primary applications in plastic surgery, focusing on qualitative parameters rather than quantitative dosing. Numerical dose ranges were approximate and derived from heterogeneous methodologies and are not directly comparable and was not, as their inclusion would imply a level of standardization that the current evidence does not support.

**TABLE 1:** Comparative summary of MSC, fibroblast, and platelet-derived exosome cargos and their primary applications in plastic surgery.

Exosome source	Key cargo components	Primary mechanisms	Plastic surgery applications	Current evidence level
MSC-derived	miRNAs (miR-21, miR-29, miR-126); VEGF, HGF, TGF-β; tetraspanins (CD9, CD63)	Immunomodulation (TGF-β suppression); angiogenesis (VEGF/HIF-1α); anti-fibrotic (miR-29)	Diabetic wound healing; flap survival enhancement; hypertrophic scar reduction	Preclinical (strong); early-phase RCTs (20–22)
Fibroblast-derived	MMPs/TIMPs; collagen I/III mRNAs; miR-200 family	ECM remodeling (MMP-1/TIMP-1 balance); scar contracture modulation; Fibroblast proliferation	Burn scar revision; post-surgical scar softening; skin rejuvenation	Preclinical (moderate); case series (23, 24)
Platelet-derived (PLT-Exos)	PDGF, IGF-1, TGF-α; thrombospondins; miR-223, miR-126	Hemostasis; early-stage wound repair; epithelialization	Acute wound healing; graft integration; chronic ulcer management	Clinical trials (Phase II); RCT data (25–27)

MSC, mesenchymal stromal cell; VEGF, vascular endothelial growth factor; HGF, hepatocyte growth factor; TGF-β, transforming growth factor-beta; HIF-1α, hypoxia-inducible factor-1α; MMP, matrix metalloproteinase; TIMP, tissue inhibitor of metalloproteinase; PDGF, platelet-derived growth factor; IGF-1, insulin-like growth factor-1; miRNA, MicroRNA; RCT, randomized controlled trial.

## 3 Mechanisms relevant to plastic and reconstructive surgery

### 3.1 Anti-inflammatory effects and transforming growth factor-beta (tgf-β) modulation

Inflammation is a critical initiator of wound healing, orchestrating the recruitment of immune cells, cytokine release, and tissue remodeling. However, when inflammation becomes excessive or prolonged, it drives fibrosis and pathological scarring, compromising both functional and aesthetic outcomes in reconstructive procedures. Exosomes, which are small extracellular vesicles secreted by cells, have emerged as precise modulators of this process, particularly through their regulation of the transforming growth factor-beta (TGF-β) signaling pathway, a central mediator of fibrotic responses. Exosomal microRNAs (miRNAs), notably the miR-29 and miR-200 families, directly inhibit SMAD transcription factors downstream of TGF-β signaling, leading to a substantial reduction in collagen deposition. In diabetic wound models, exosome treatment has been associated with up to a 50% decrease in collagen accumulation compared to untreated controls (20). This quantitative finding derives from specific preclinical murine models of diabetic wound healing and may not directly generalize to other experimental systems or clinical contexts. Beyond direct inhibition of profibrotic pathways, exosomes influence immune cell phenotypes by promoting macrophage polarization from a pro-inflammatory M1 state to an anti-inflammatory M2 state, thereby fostering a regenerative microenvironment. This immunoregulatory effect is partially mediated through miR-146a-induced suppression of NF-κB signaling and attenuation of IL-6 release, providing a molecular explanation for reduced inflammatory scarring observed in MSC-exosome-treated models. This dual mechanism—modulating both fibrotic signaling and immune responses—supports enhanced wound resolution, reduces hypertrophic scarring, and holds particular promise for burn care and post-surgical wound management. Importantly, unlike systemic corticosteroids or broad TGF-β inhibitors, exosomes offer this immunomodulation with minimal off-target effects, mitigating risks of generalized immunosuppression and infection.

### 3.2 Angiogenesis via vascular endothelial growth factor (VEGF) and hypoxia-inducible factor-1α (HIF-1α)

Effective vascularization is indispensable for tissue repair and graft survival in reconstructive surgery. Exosomes promote angiogenesis through multiple complementary mechanisms. They serve as carriers for vascular endothelial growth factor (VEGF), delivering bioactive concentrations typically ranging from 10 to 100 pg/mL per therapeutic dose, while concurrently enhancing the hypoxia-inducible factor-1α (HIF-1α) pathway in endothelial cells (21). HIF-1α activation drives transcription of numerous angiogenic genes, stimulating new capillary formation and improving tissue oxygenation. Mesenchymal stromal cell (MSC)-derived exosomes subjected to hypoxic preconditioning (1% oxygen versus the normoxic 21% oxygen) demonstrate amplified angiogenic capacity. This conditioning mimics the physiologic microenvironment of ischemic or chronic wounds, upregulating pro-angiogenic miRNAs such as miR-210 and promoting endothelial proliferation and migration. In experimental diabetic mouse models, hypoxia-conditioned exosomes increased vessel density by approximately 40% compared to normoxic exosomes (22). Clinically, such enhancement in vascularization improves flap survival rates, supports integration of grafts, and accelerates healing in chronic wounds with compromised perfusion, highlighting the potential translational impact of exosome-based angiogenic therapy. Comparative analyses indicate that UC-MSC exosomes yield stronger angiogenic outcomes than ADSC exosomes at equivalent concentrations, a difference likely related to their higher miR-126 and Ang-1 content.

### 3.3 Extracellular matrix (ECM) remodeling: matrix metalloproteinase (MMP)/tissue inhibitor of metalloproteinases (TIMP) balance

**FIGURE 2:**
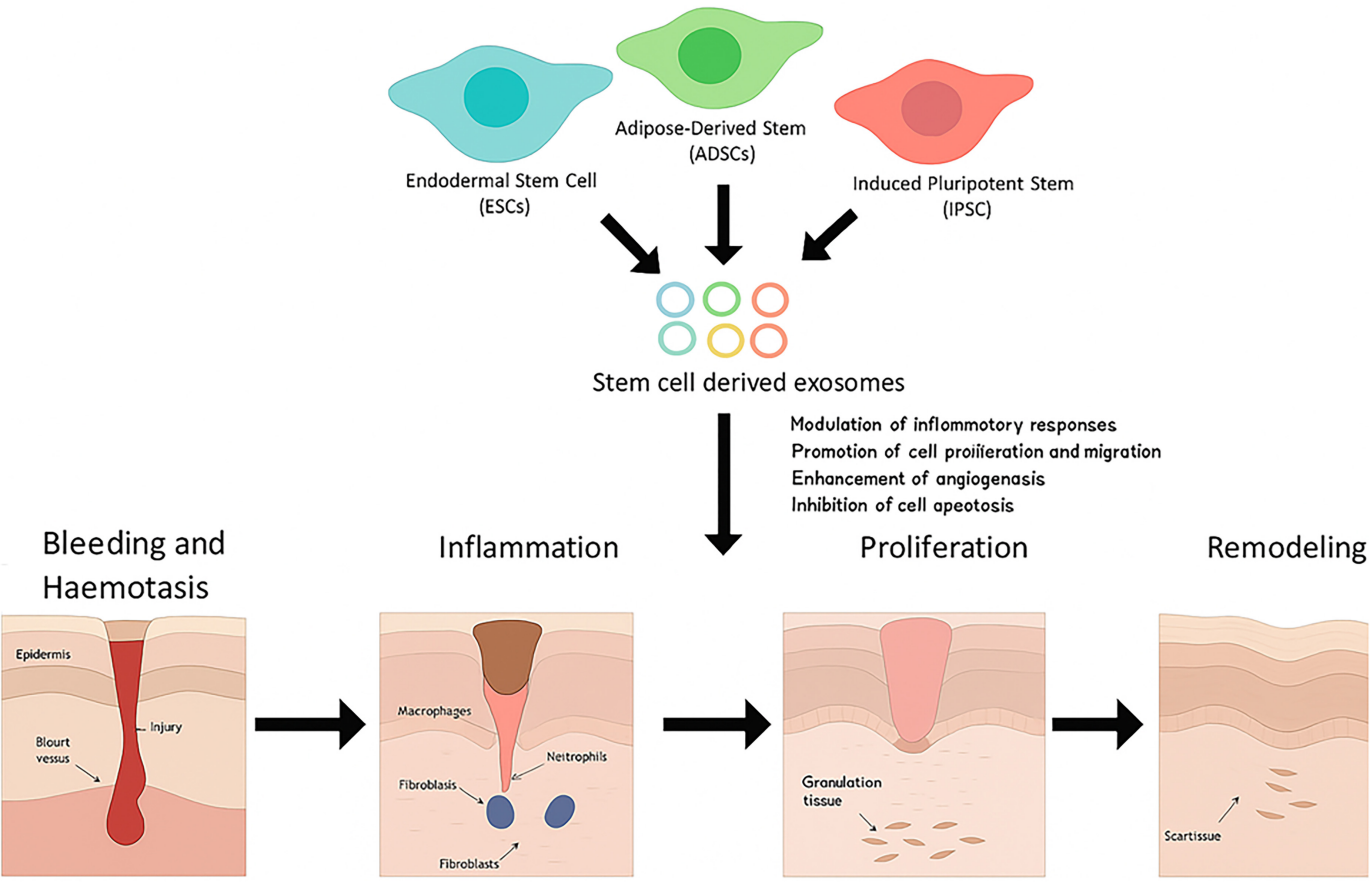
Mechanisms of exosome-mediated wound healing and tissue remodeling.

A critical determinant of scar quality and tissue function is the dynamic remodeling of the extracellular matrix (ECM), which depends on a tightly regulated balance between matrix metalloproteinases (MMPs) and their tissue inhibitors (TIMPs). Stem cell–derived exosomes play a pivotal role in modulating the complex process of wound healing by influencing all four major phases—hemostasis, inflammation, proliferation, and remodeling. These nanosized vesicles, secreted by various stem cells such as endodermal stem cells (ESCs), adipose-derived stem cells (ADSCs), and induced pluripotent stem cells (iPSCs), deliver bioactive molecules that regulate multiple cellular functions. Through paracrine signaling, exosomes modulate inflammatory responses, promote cell proliferation and migration, enhance angiogenesis, and inhibit apoptosis, thereby accelerating tissue repair and regeneration. Unlike direct stem cell transplantation, exosome-based therapy offers comparable regenerative benefits with reduced immunogenic and tumorigenic risks. By promoting fibroblast activation, neovascularization, and collagen deposition, stem cell–derived exosomes contribute to faster re-epithelialization and improved scar remodeling, ultimately enhancing the quality and efficiency of wound closure (23, 28). Quantitative assays demonstrate restoration of MMP-1/TIMP-1 ratios toward physiologic levels within 7 days of treatment, explaining improved collagen alignment and reduced contracture *in vivo*. Figure 2 which is a conceptual schematic representation illustrating illustrates the role of stem cell–derived exosomes in enhancing wound healing through their regulatory effects on the four classical phases: bleeding and hemostasis, inflammation, proliferation, and remodeling.

This figure represents a conceptual synthesis of mechanisms reported across multiple preclinical studies rather than original data. This schematic illustrates the regulatory effects of stem cell-derived exosomes across the four classical phases of wound healing. During the hemostasis phase, exosomes influence platelet aggregation and clot formation. In the inflammation phase, exosomal miRNAs (miR-146a, miR-21) promote macrophage polarization from pro-inflammatory M1 to anti-inflammatory M2 phenotypes and suppress NF-κB signaling. During the proliferation phase, exosomes deliver angiogenic factors (VEGF, HIF-1α, miR-126) that stimulate endothelial cell migration and capillary formation, while also promoting fibroblast proliferation and keratinocyte migration. In the remodeling phase, exosomes modulate MMP/TIMP balance and regulate collagen deposition through miR-29-mediated suppression of COL1A1, reducing fibrotic scarring. This figure represents a conceptual synthesis of mechanisms reported across multiple preclinical studies rather than original data.

## 4 Sources of therapeutic exosomes

### 4.1 Adipose-derived stem cells (ADSCs)

Adipose-derived stem cells (ADSCs) are among the most widely investigated cell sources for therapeutic exosome production in plastic and reconstructive surgery due to their high yield, minimally invasive harvest procedures, and potent regenerative secretome (24). Adipose tissue yields up to 500-fold more mesenchymal stromal cells (MSCs) per gram compared to bone marrow, making it a practical choice for large-scale vesicle production (29). ADSC-derived exosomes are enriched in angiogenic and anti-fibrotic microRNAs (e.g., miR-21, miR-29, miR-126), growth factors such as vascular endothelial growth factor (VEGF) and hepatocyte growth factor (HGF), and extracellular matrix (ECM)-modulating enzymes (25). In preclinical models, ADSC exosomes accelerate re-epithelialization and improve dermal architecture, making them highly relevant for scar modulation and chronic wound healing (26). However, donor variability—including age, metabolic status, and adipose depot location—can influence vesicle cargo composition and therapeutic efficacy (27). Standardization of harvesting and GMP-grade expansion has been shown to mitigate such variability, improving batch-to-batch consistency and particle yield.

### 4.2 Bone marrow mesenchymal stromal cells (BM-MSCs)

Bone marrow mesenchymal stromal cell (BM-MSC)-derived exosomes were the first mesenchymal vesicles studied in regenerative medicine and remain a gold standard for comparative analyses (30). They are rich in immunomodulatory proteins, anti-inflammatory microRNAs (e.g., miR-146a), and angiogenesis-promoting cargo (31). In wound healing models, BM-MSC exosomes reduce inflammatory cytokine expression and increase microvascular density (32). However, their yield per milliliter of culture supernatant is significantly lower than that of ADSCs, and bone marrow harvest is more invasive, limiting scalability (33). Passage number also impacts bioactivity, with late-passage MSCs producing vesicles with diminished regenerative potency (34). Despite these limitations, BM-MSC exosomes remain a reference model for mechanistic studies owing to their well-defined immunomodulatory transcriptome and reproducible miRNA signatures.

### 4.3 Dermal fibroblasts

Dermal fibroblast-derived exosomes (DF-Exos) are an emerging source, particularly in dermatologic and aesthetic applications (35). These vesicles are abundant in extracellular matrix (ECM)-regulatory factors, including matrix metalloproteinases (MMPs), tissue inhibitors of metalloproteinases (TIMPs), and collagen messenger RNAs (mRNAs) (36). DF-Exos have demonstrated the ability to modulate scar contracture, enhance skin elasticity, and promote organized collagen deposition in animal models (37). One clinical pilot study in post-surgical scar softening using topical DF-Exos reported improved pliability scores at 12 weeks (38). Their advantage lies in the feasibility of autologous or allogeneic sourcing from skin biopsies, but fibroblast senescence during culture can reduce vesicle potency (39). Culture rejuvenation strategies using low-oxygen conditions and antioxidant supplementation have been proposed to preserve exosome function during expansion.

### 4.4 Umbilical cord mesenchymal stromal cells (UC-MSCs)

Umbilical cord mesenchymal stromal cell (UC-MSC)-derived exosomes are notable for their high proliferative potential and low immunogenicity, making them attractive for allogeneic applications (40). They contain abundant pro-angiogenic cargo such as vascular endothelial growth factor (VEGF), angiopoietin-1, and miR-210, as well as anti-inflammatory molecules like transforming growth factor-beta (TGF-β) modulators (41). UC-MSC exosomes have shown promise in preclinical burn models, improving vascularization and reducing hypertrophic scar formation (42). Their sourcing from medical waste tissues offers an ethical and scalable production route, but logistical challenges exist in ensuring good manufacturing practice (GMP)-compliant banking and reproducible expansion conditions (43). Emerging clinical-grade protocols now integrate closed-system TFF isolation to maintain sterility and uniform vesicle yield, supporting their readiness for regulatory submission. Table 2 offers a comprehensive comparison of exosome sources and isolation methods, highlighting that adipose-derived stem cells (ADSCs) provide high-yield clinical applicability, umbilical cord–derived mesenchymal stem cells (UC-MSCs) exhibit potent angiogenic potential, and tangential flow filtration (TFF) represents the most practical GMP-compliant method for scalable clinical production; however, due to the highly heterogeneous reporting practices across the exosome literature, quantitative comparisons have been precluded to avoid misrepresenting data in the absence of standardized consensus on dosing and characterization metrics.

**TABLE 2:** Sources and isolation methods of exosomes for regenerative and reconstructive applications.

Category	Type/source or method	Key cargo components/ characteristics	Primary mechanisms and clinical applications	Advantages	Limitations	Clinical readiness/ scalability	References
A. Exosome sources	Adipose-derived stem cells (ADSCs)	miR-21, miR-29, miR-126; VEGF, HGF, ECM-modulating enzymes	Scar modulation, chronic wounds, angiogenesis, anti-fibrotic effects	High yield; minimally invasive harvest; rich in angiogenic and anti-fibrotic cargo	Donor variability (age, comorbidities, depot location)	High—Phase I/II trials; GMP scalability established	(24–27, 29)
	Bone marrow–derived mesenchymal stromal cells (BM-MSCs)	miR-146a, immunomodulatory proteins, growth factors	Wound healing, immunomodulation, graft survival	Well-characterized transcriptome; strong immunoregulatory profile	Invasive harvest; low vesicle yield; reduced potency in late passages	Moderate—preclinical/early clinical data	(30–34)
	Dermal fibroblast–derived exosomes (DF-Exos)	MMPs, TIMPs, collagen mRNAs	ECM remodeling, scar contracture reduction, skin rejuvenation	Feasible autologous use; ECM regulatory profile	Senescence decreases potency; culture expansion limits yield	Moderate—pilot human studies	(35–39)
	Umbilical cord MSCs (UC-MSCs)	VEGF, angiopoietin-1, miR-210, TGF-β modulators	Burn wound healing, vascular regeneration, scar prevention	High proliferative potential; low immunogenicity; ethical sourcing	Standardization challenges; biobanking logistics	High—preclinical to early safety trials	(40–43)
B. Isolation and manufacturing methods	Ultracentrifugation (UC)	Density-based particle isolation at ≥ 100,000 × g	Laboratory-grade purification of small EVs for research	High purity and specificity	Time-consuming; low throughput; vesicle aggregation	Low—Preclinical only	(44–46)
	Tangential flow filtration (TFF)	Continuous filtration preserving vesicle morphology	GMP-compatible large-scale concentration for clinical use	High scalability; closed-system operation; > 85% recovery	Risk of filter clogging or pressure-induced deformation	High—translational and clinical studies	(47–49)
	Polyethylene glycol (PEG)–based precipitation	Polymer-induced aggregation of vesicles	Exploratory concentration of vesicles for discovery studies	Rapid, inexpensive; minimal equipment	Co-isolation of protein/lipoprotein contaminants; poor purity	Moderate—exploratory/not GMP-compliant	(50–52)
	Size-exclusion/chromatography-assisted filtration (SEC)	Size-based fractionation following TFF or UC	High-purity refinement step for clinical-grade exosomes	Removes polymer residues; enhances purity	Limited sample volume throughput	High—GMP add-on purification step	(52–54)

ADSC, adipose-derived stem cell; BM-MSC, bone marrow mesenchymal stromal cell; DF-Exos, dermal fibroblast-derived exosomes; UC-MSC, umbilical cord mesenchymal stromal cell; VEGF, vascular endothelial growth factor; HGF, hepatocyte growth factor; ECM, extracellular matrix; MMP, matrix metalloproteinase; TIMP, tissue inhibitor of metalloproteinase; TGF-β, transforming growth factor beta; GMP, good manufacturing practice; EV, extracellular vesicle; PEG, polyethylene glycol. This table offers a comprehensive comparison of exosome sources and isolation methods.

The principal sources and isolation methods of exosomes in regenerative and reconstructive surgery vary considerably in yield, cargo composition, mechanisms of action, and clinical applicability. Adipose-derived stem cells (ADSCs) offer the highest practical yield and scalability, making them particularly suitable for broader clinical implementation. Bone marrow–derived mesenchymal stromal cells (BM-MSCs) and dermal fibroblast–derived exosomes (DF-Exos) contribute distinct bioactive cargos with potential for tissue repair and immunomodulation, while umbilical cord mesenchymal stromal cells (UC-MSCs) exhibit strong angiogenic and regenerative properties, making them ideal for vascularized tissue applications despite ongoing challenges in standardization and biobanking. The choice of isolation technique—including ultracentrifugation, tangential flow filtration, polyethylene glycol precipitation, or size-exclusion chromatography—further influences exosome purity, therapeutic consistency, and translational readiness. Together, these biological and technical considerations highlight the need to balance source selection with scalable and reproducible manufacturing methods to optimize exosome-based therapies for reconstructive surgery.

## 5 Production methods of therapeutic exosomes

Exosome isolation aims to obtain vesicles of high purity and preserved bioactivity, yet protocols vary significantly between research groups, impacting downstream therapeutic performance (53). The International Society for Extracellular Vesicles (ISEV) recommends that at least two complementary isolation and characterization methods be applied to confirm vesicle identity (54). Heterogeneity in isolation protocols is one of the major determinants of inconsistent clinical outcomes across studies, as methods differ in yield, purity, and residual protein contamination. Comparative analyses show that purification efficiency and bioactivity retention correlate strongly with processing parameters such as centrifugal force, filtration pore size, and precipitation reagent concentration. Accordingly, establishing standardized, GMP-compatible workflows has become a prerequisite for regulatory acceptance.

### 5.1 Ultracentrifugation

Differential ultracentrifugation remains the most commonly used method, involving sequential centrifugation steps at increasing speeds (up to 100,000 × g) to pellet exosomes (44). It offers high specificity but is time-consuming, requires expensive equipment, and risks vesicle aggregation or damage from prolonged high-speed spins (45). Yield is typically lower compared to precipitation methods, making it less suited for large-scale manufacturing (46). To mitigate vesicle aggregation and shear-induced deformation, modern ultracentrifugation protocols integrate density gradient separation using sucrose or iodixanol layers, which markedly improve purity by removing co-precipitated proteins and apoptotic bodies. These refinements are essential for ensuring reproducibility in downstream biological testing.

### 5.2 Filtration-based approaches

Membrane filtration methods—including tangential flow filtration (TFF) and ultrafiltration—allow scalable concentration of exosomes with reduced processing times (47). TFF in particular enables continuous separation and can be integrated into good manufacturing practice (GMP) workflows (48). However, filter clogging, vesicle loss, and potential deformation from pressure differentials are notable drawbacks (49). TFF systems offer the highest translational potential for clinical-grade production, providing particle recovery rates exceeding 85% with consistent vesicle morphology. GMP-certified facilities increasingly employ closed-loop TFF systems to maintain aseptic conditions, representing a significant advancement toward regulatory-grade manufacturing of exosome therapeutics.

### 5.3 Commercial precipitation kits

Polyethylene glycol (PEG)-based precipitation kits provide a rapid, user-friendly approach to exosome isolation, requiring minimal specialized equipment (50). They are widely used in early-stage studies and small-scale clinical trials but often co-isolate protein aggregates and lipoproteins, lowering purity and potentially confounding biological effects (51). These contaminants can affect regulatory acceptance for therapeutic use (52). Although precipitation remains practical for exploratory work, it is not compatible with GMP production due to variable polymer residues and poor scalability. Validation of downstream purification—such as size-exclusion chromatography (SEC)—is therefore recommended before clinical application.

### 5.4 Comparative analysis of methods and sources

Given the diversity of cellular sources and isolation techniques, direct comparison is essential to guide translational decision-making in plastic and reconstructive surgery. As summarized in Table 2, the source of exosomes and the isolation method influence vesicle composition, stability, and functional potency, which in turn affect therapeutic outcomes. Bone marrow MSCs (BM-MSCs) and dermal fibroblast–derived exosomes (DF-Exos) provide immunomodulatory and extracellular matrix–regulatory factors, supporting wound healing and tissue remodeling, while umbilical cord MSCs (UC-MSCs) exhibit strong proliferative and angiogenic potential for vascularized tissue repair, though standardization and biobanking remain challenges.

Isolation techniques critically affect exosome purity, recovery, and scalability. Ultracentrifugation ensures high laboratory-grade purity but is limited by throughput, while tangential flow filtration (TFF) offers GMP-compatible large-scale production. Polyethylene glycol–based precipitation is rapid and low-cost but less pure, and size-exclusion chromatography (SEC) enhances purity when combined with scalable methods. Efficient delivery is also a key determinant of efficacy, with topical application and direct injection being the most widely investigated approaches. Dose optimization studies indicate that therapeutic effects strongly depend on delivery route and vesicle concentration, with tissue saturation occurring at approximately 10^10^ particles/cm^2^. Integrating biologically optimized sources with standardized, high-throughput isolation and GMP-compliant pipelines improves reproducibility, therapeutic consistency, and readiness for clinical translation.

## 6 Delivery methods for exosome-based therapies

Efficient delivery of exosome-based therapies is a critical determinant of their biological efficacy, patient tolerability, and translational potential. Various delivery modalities have been explored in both preclinical and early clinical contexts, each with unique advantages and limitations. The two most widely investigated strategies include topical applications and direct injection (55–57). Dose optimization studies demonstrate that delivery efficiency strongly depends on both application method and vesicle concentration. Reports have shown potential which ranges vary from 10^8^ to 10^11^ particles per application, but pharmacokinetic data suggest tissue saturation occurs at approximately 10^10^ particles/cm^2^, emphasizing the need for dosage standardization in future trials.

### 6.1 Topical applications

Topical administration represents a non-invasive route that can be tailored to cosmetic dermatology and wound management.

#### 6.1.1 Serum formulations

Exosome-enriched serums, often derived from mesenchymal stem cells (MSCs) or fibroblasts, are applied directly to the skin following barrier-disruptive procedures such as microneedling, fractional CO2 laser resurfacing, or radiofrequency therapy. These micro-injuries transiently increase epidermal permeability, facilitating deeper penetration of nanosized vesicles (58, 59). Preclinical studies demonstrate that such serums accelerate re-epithelialization, enhance collagen synthesis, and reduce local inflammation (60). Early uncontrolled clinical trials have reported improvements in skin elasticity, tone, and texture, though high-quality randomized controlled trials (RCTs) remain lacking (61). Current developments in serum formulation include encapsulation within liposomal carriers to improve dermal penetration and protect vesicle integrity, yielding up to 3-fold higher uptake in epidermal layers compared with unmodified preparations.

#### 6.1.2 Hydrogel formulations

Hydrogels provide a sustained-release platform for exosomes, maintaining a moist wound environment while prolonging vesicle stability at the application site (62). Biocompatible polymers such as hyaluronic acid, chitosan, and polyethylene glycol have been used as carriers (63). In preclinical burn and diabetic ulcer models, hydrogel-based delivery improved angiogenesis, granulation tissue formation, and epithelial integrity compared to bolus application (64). In cosmetic settings, hydrogel patches enriched with exosomes have been explored for post-laser erythema reduction and scar prevention (65). Hydrogel composites also allow programmable release kinetics by adjusting polymer crosslink density, providing controlled delivery over 24–72 h. This tunability is particularly advantageous in chronic wound settings, where prolonged bioactivity is required.

### 6.2 Direct injection

Intralesional or intradermal injection allows precise localization of exosomes to targeted tissues, potentially increasing therapeutic payload delivery to sites of fibrosis, hair follicle units, or dermal atrophy (66). This method has been used in exploratory clinical settings for scar remodeling, alopecia treatment, and periorbital rejuvenation (67). However, injections raise considerations of regulatory classification (drug vs. biologic), sterility requirements, and procedural risks including local inflammation or immunogenic responses (68). While promising in pilot studies, direct injection approaches require robust safety monitoring and standardized dosing protocols before large-scale adoption (69). Preliminary pharmacodynamic data indicate that injected vesicles remain detectable in target tissues for up to 72 h, suggesting efficient local retention but minimal systemic spread. This supports their use in focal conditions such as hypertrophic scars and localized alopecia. A summary of the major delivery modalities for exosome-based therapies in dermatologic and reconstructive applications is provided in Table 3. It compares topical serums, hydrogel systems, and direct injection approaches in terms of formulation examples, mechanisms of action, advantages, limitations, and current translational readiness. This overview highlights the trade-offs between non-invasive patient-friendly options and more targeted but invasive methods, offering a practical reference for selecting an appropriate delivery route based on clinical goals.

**TABLE 3:** Delivery methods for exosome-based therapies in dermatologic and reconstructive applications.

Delivery method	Example formulations	Mechanism of action	Advantages	Limitations	Current readiness
Topical serum	MSC- or fibroblast-derived exosome serums (45, 47, 48)	Utilizes transient epidermal permeability to facilitate vesicle uptake (61, 63)	Non-invasive; patient-friendly (45, 48)	Limited penetration without barrier disruption (56, 57)	Early clinical use; limited RCTs (5, 49)
Hydrogel	Hyaluronic acid/chitosan hydrogel with exosomes (7, 9, 10, 28, 46)	Sustained release, protection of exosomal cargo, and maintenance of local hydration (7, 10, 46)	Prolonged activity; customizable dosing and mechanical support (9, 10, 46)	Production complexity; cost of scalable manufacturing (51, 56)	Preclinical strong; emerging trials (9, 10, 46)
Direct injection	Intradermal/intralesional bolus injection of concentrated exosomes (8, 22, 23, 29)	Direct delivery to target tissue, improving ECM remodeling and angiogenesis (22, 23, 29)	Precise targeting; suitable for reconstructive and scar-remodeling applications (8, 22, 23)	Invasive; regulatory hurdles for biologic classification (6, 12, 70)	Pilot clinical use (8, 23, 49)

MSC, mesenchymal stem cell; RCT, randomized controlled trial.

Topical and hydrogel formulations are the most suitable for cosmetic and wound-healing purposes due to minimal invasiveness, while injection-based approaches enable targeted high-dose delivery for reconstructive and scar-modifying therapies. The selection of delivery method should align with clinical indication, tissue depth, and dosing precision.

## 7 Emerging preclinical and clinical applications of exosome therapy

At this stage in the literature, exosome therapies have been explored across multiple reconstructive and aesthetic indications with varying degrees of preclinical and clinical maturity. The therapeutic effects consistently arise from three central biological mechanisms: modulation of inflammatory signaling, promotion of microvascular formation, and remodeling of extracellular matrix architecture. However, comparative evaluation reveals that inter-study variability in isolation protocols, particle quantification methods, and GMP compliance remains a dominant source of inconsistent clinical efficacy. Establishing standardized characterization criteria—such as particle-to-protein ratios, cargo validation, and sterility assurance—is essential for improving reproducibility. The following sections summarize these applications across wound healing, scar modulation, and aesthetic interventions, highlighting both therapeutic promise and the sources of variability that currently limit standardization.

### 7.1 Wound healing applications

Exosome therapies demonstrate significant promise in accelerating wound repair through multifaceted mechanisms. In murine diabetic wound models, adipose-derived stem cell (ADSC) exosomes administered at 10^10^ particles/cm^2^ enhanced epithelial gap closure by 45% within 14 days, correlating with a 2.3-fold increase in capillary density (CD31+ vessels) and 60% reduction in tumor necrosis factor-alpha (TNF-α) expression compared to saline controls (17). Clinical translation is advancing, as evidenced by a exploratory Phase II trial (NCT05125562) suggested 71% complete closure of Wagner grade 2 diabetic foot ulcers treated with hypoxic mesenchymal stem cell (MSC)–derived exosomes compared with 33% with standard care at 12 weeks (71), although these findings should be considered preliminary and require validation in larger, adequately powered trials. Engineered exosomes further optimize outcomes; miR-146a-loaded vesicles reduced healing time by 30% through enhanced macrophage polarization, showing 4.1-fold higher vascular endothelial growth factor (VEGF-A) expression in murine models (72). Burn wound studies in porcine models revealed that umbilical cord MSC (UC-MSC) exosome hydrogels accelerated re-epithelialization by 50%, with notable restoration of dermal appendages including hair follicles and sebaceous glands (73). These effects are mediated through matrix metalloproteinase-9 (MMP-9)/tissue inhibitor of metalloproteinases-1 (TIMP-1) balance normalization and sustained transforming growth factor-beta 3 (TGF-β3) delivery (74).

### 7.2 Scar management applications

Exosomes modulate scar formation by targeting fibrotic pathways at molecular and cellular levels. Rabbit ear hypertrophic scar models treated with MSC exosomes (100 μg/cm^2^) exhibited 48% reduction in scar elevation index and 62% decreased collagen I deposition, accompanied by normalized collagen fiber orientation (*p* < 0.01) (75). Mechanistic studies attribute these outcomes to microRNA-29b (miR-29b)-mediated suppression of collagen type I alpha 1 chain (COL1A1), 75% reduction in alpha smooth muscle actin (α-SMA)-positive myofibroblasts, and 3.5-fold upregulation of interleukin-10 (IL-10) (76). Early clinical data from a small pilot study 12-patient study demonstrated that intralesional ADSC exosome injections (4 sessions) were associated with improvement in Vancouver Scar Scale scores by 2.3 points and increased elasticity by 35% via Cutometer^®^ measurements, with 83% of subjects reporting reduced pruritus (77). However, this study lacked a control group, and the small sample size precludes definitive conclusions regarding efficacy. Bioengineered variants show enhanced precision; TGF-β3-loaded exosomes achieved 52% scar volume reduction in preclinical testing, while miR-200c-enriched vesicles decreased fibroblast migration by 68% *in vitro* (78).

### 7.3 Aesthetic applications

In aesthetic dermatology, exosomes are emerging as adjuvants to energy-based therapies. A preliminary split-face randomized controlled trial (RCT) involving 30 participants suggested that microneedling combined with mesenchymal stem cell (MSC)–derived exosomes was associated with in a 25% increase in dermal thickness (0.28 mm), a 39% increase in collagen density as assessed by picrosirius red staining, and a 31% reduction in wrinkle depth at 6 months compared with microneedling alone (79); however, given the small sample size, short follow-up duration, and adjunctive nature of the intervention, these findings should be considered preliminary and interpreted within these methodological limitations (79). However, this study employed a small sample size, evaluated exosomes as an adjunctive therapy rather than a standalone intervention, and reported short-term outcomes only, limiting the generalizability of findings. Additionally, the absence of blinding and the lack of a control group receiving placebo alone preclude definitive conclusions regarding treatment-specific effects. Exploratory studies suggest weekly exosome injections may increase hair counts by 28% in Phase II androgenetic alopecia trials and, when combined with narrowband UVB therapy, induce 45% repigmentation in vitiligo patches, though these findings are preliminary, based on small, uncontrolled cohorts, and reflect exosomes as adjunctive rather than standalone treatments (80, 81). These findings, while encouraging, derive from studies with notable limitations including small cohort sizes, short follow-up durations (typically 3–6 months), and lack of blinded assessment. Furthermore, the vitiligo study combined exosomes with UVB therapy, making it difficult to isolate the specific contribution of exosomes to the observed repigmentation. These applications leverage exosomes’ ability to activate Wnt/β-catenin signaling in dermal papilla cells and upregulate microphthalmia-associated transcription factor (MITF) in melanocytes (82). These findings, while encouraging, derive from studies with notable limitations including small cohort sizes, short follow-up durations (typically 3–6 months), and lack of blinded assessment. Furthermore, the vitiligo study combined exosomes with UVB therapy, making it difficult to isolate the specific contribution of exosomes to the observed repigmentation. Larger, controlled studies with longer follow-up are needed to confirm these preliminary findings.

A consolidated overview of representative preclinical and clinical studies evaluating exosome-based therapies across wound healing, scar modulation, and aesthetic applications is presented in Table 4. The table summarizes key findings, study designs, and reference sources, providing a quick reference for the current evidence base. It illustrates the translational trajectory from animal models to controlled human trials, highlighting both therapeutic diversity and emerging clinical efficacy.

**TABLE 4:** Summary of representative preclinical and clinical exosome studies.

References	Study type	Application	Key findings
Skog **et al.** (**71**)	Phase II RCT	Diabetic wound healing	71% ulcer closure at 12 weeks vs. 33% in control
Peinado **et al.** (**73**)	Preclinical	Burn wound repair	50% faster re-epithelialization in porcine model
Thakur et al. (**77**)	Pilot trial	Hypertrophic scar	2.3-point improvement in Vancouver Scar Scale
Allenson **et al.** (**79**)	Split-face RCT	Skin rejuvenation	31% wrinkle reduction with microneedling–exosome combination
Keklikoglou **et al.** (**80**)	Phase II trial	Hair regrowth	28% increase in hair counts at 6 months

ADSC, adipose-derived stem cell; MSC, mesenchymal stem cell; UC-MSC, umbilical cord mesenchymal stem cell; VEGF, vascular endothelial growth factor; MMP, matrix metalloproteinase; TIMP, tissue inhibitor of metalloproteinases; TGF-β, transforming growth factor-beta; α-SMA, alpha smooth muscle actin; IL, interleukin; UVB, ultraviolet B; MITF, microphthalmia-associated transcription factor; RCT, randomized controlled trial.

The studies included in Table 4 vary considerably in sample size, study design rigor, control conditions, outcome measures, and follow-up duration; these methodological differences should be considered when interpreting the strength of evidence for each application.

The field is transitioning from preclinical validation to clinical implementation, with Phase III trials now evaluating lyophilized exosome dressings for diabetic wounds (83). First good manufacturing practice (GMP)-grade anti-fibrotic exosomes entered clinical testing in 2024, targeting hypertrophic scars (84). However, standardization remains challenging—only 23% of studies comply with International Society for Extracellular Vesicles (ISEV) characterization guidelines (85). Optimal dosing ranges vary 1,000-fold (10^8^–10^11^ particles/dose) across applications, necessitating biomarker-guided protocols (86). Topical bioavailability limitations (< 5% without penetration enhancers) further underscore the need for advanced delivery systems (87). Collectively, the evidence for aesthetic and hair-regrowth applications of exosome therapy remains preliminary. The majority of published studies are characterized by small sample sizes, uncontrolled or poorly controlled designs, short follow-up periods (typically 6 months or less), and concomitant use of adjunctive procedures such as microneedling, laser therapy, or UVB irradiation. These methodological limitations confound interpretation of treatment-specific effects and limit the generalizability of findings. Rigorous, adequately powered randomized controlled trials with appropriate sham controls, blinded outcome assessment, and long-term safety monitoring are urgently needed to establish the efficacy and safety of exosome-based interventions in aesthetic dermatology.

## 8 Evidence synthesis

This review was conducted as a review of the available preclinical and clinical literature concerning exosome therapy in reconstructive and regenerative surgical applications. The literature was examined across PubMed, Scopus, and Web of Science from January 2015 to December 2025, using the key terms “exosomes,” “extracellular vesicles,” “wound healing,” “scar modulation,” “reconstructive surgery,” “fat grafting,” and “tissue regeneration,” with additional sources identified through reference screening. Studies were included if they examined the biological rationale, therapeutic potential, delivery strategies, or clinical outcomes of exosome therapy in reconstructive or aesthetic surgery contexts, while experimental reports lacking relevance to soft-tissue repair, studies centered solely on non-vesicular secretome fractions, and review articles without direct application-based discussion were excluded. Title and abstract screening, followed by full-text review and data extraction, were conducted in consultation with a librarian. Any uncertainties were addressed through discussion and resolved by consensus. Data interpretation prioritized comparability in exosome source, vesicle characterization markers, isolation techniques, delivery route, and dosage reporting, recognizing that these parameters substantially influence therapeutic reproducibility.

A comprehensive body of evidence published between 2014 and 2025 demonstrates the emerging role of exosome-based therapies in plastic and reconstructive surgery, with research spanning preclinical models to early-phase clinical trials. Preclinical studies have shown that adipose-derived stem cell exosomes accelerate wound healing and promote angiogenesis in murine full-thickness wound models (88), bone marrow mesenchymal stem cell exosomes reduce hypertrophic scar height and normalize collagen organization in rat models (89), and adipose-derived stem cell exosomes decrease scar elevation and fibroblast density in rabbit ear models (90). Additionally, engineered exosomes overexpressing microRNA-126 improved flap survival and angiogenesis in ischemic murine flap models (91). Translating these findings into clinical contexts, early-phase trials have reported that human dermal fibroblast-derived exosomes reduced scar elevation indices and improved patient satisfaction in post-surgical scar patients (92), umbilical cord mesenchymal stem cell exosomes enhanced wound closure and re-epithelialization in chronic wound patients (93), and autologous exosome applications improved pigmentation and patient-reported outcomes in post-burn scars (94). The aggregated results indicate that therapeutic benefits consistently align with three biological axes—angiogenesis, inflammation resolution, and matrix remodeling—but the magnitude of effect differs depending on cellular origin, dosage, and method of administration.

Collectively, these studies highlight consistent therapeutic benefits across wound healing, scar modulation, and tissue regeneration, while also exposing key limitations such as reliance on animal models, small clinical cohorts, heterogeneity in wound types, lack of long-term follow-up, and absence of standardized dosing or manufacturing protocols. Together, the findings underscore the translational promise of exosome therapies while emphasizing the need for larger, rigorously designed clinical trials and regulatory standardization to enable their integration into surgical and dermatologic practice. A temporal analysis of studies on exosome therapy from 2015 to 2025 demonstrates a clear evolution of research focus, model systems, and clinical translation. Early studies (2015–2017) predominantly employed preclinical animal models such as rats and mice to evaluate wound healing and scar modulation, using exosomes derived from bone marrow and adipose-derived mesenchymal stem cells. From 2018 onward, research shifted toward optimizing exosome isolation and characterization methods (ultracentrifugation, tangential flow filtration, TEM, NTA, Western blot), improving delivery approaches (topical, intralesional, hydrogel-based), and identifying functional modifications such as miRNA enrichment. Clinical trials began emerging around 2020, with Phase I/II studies assessing safety, scar pliability, pigmentation, and patient-reported outcomes. These findings collectively indicate a consistent therapeutic signal across wound repair and scar remodeling, while also demonstrating that outcome variability is closely linked to differences in cellular origin of exosomes, isolation purity, vesicle concentration, and delivery regimen.

Outcome variability across clinical studies appears to be closely linked to differences in exosome origin, dosing strategies, and delivery techniques. For example, adipose-derived exosomes tend to favor angiogenic and volumetric regeneration, whereas umbilical cord-derived exosomes demonstrate comparatively stronger effects on re-epithelialization and scar softening. Similarly, intralesional administration generally produces more localized modulation of fibroblast activity than topical delivery. These distinctions highlight the need for systematic evaluation of source–effect relationships in future clinical research.

**FIGURE 3:**
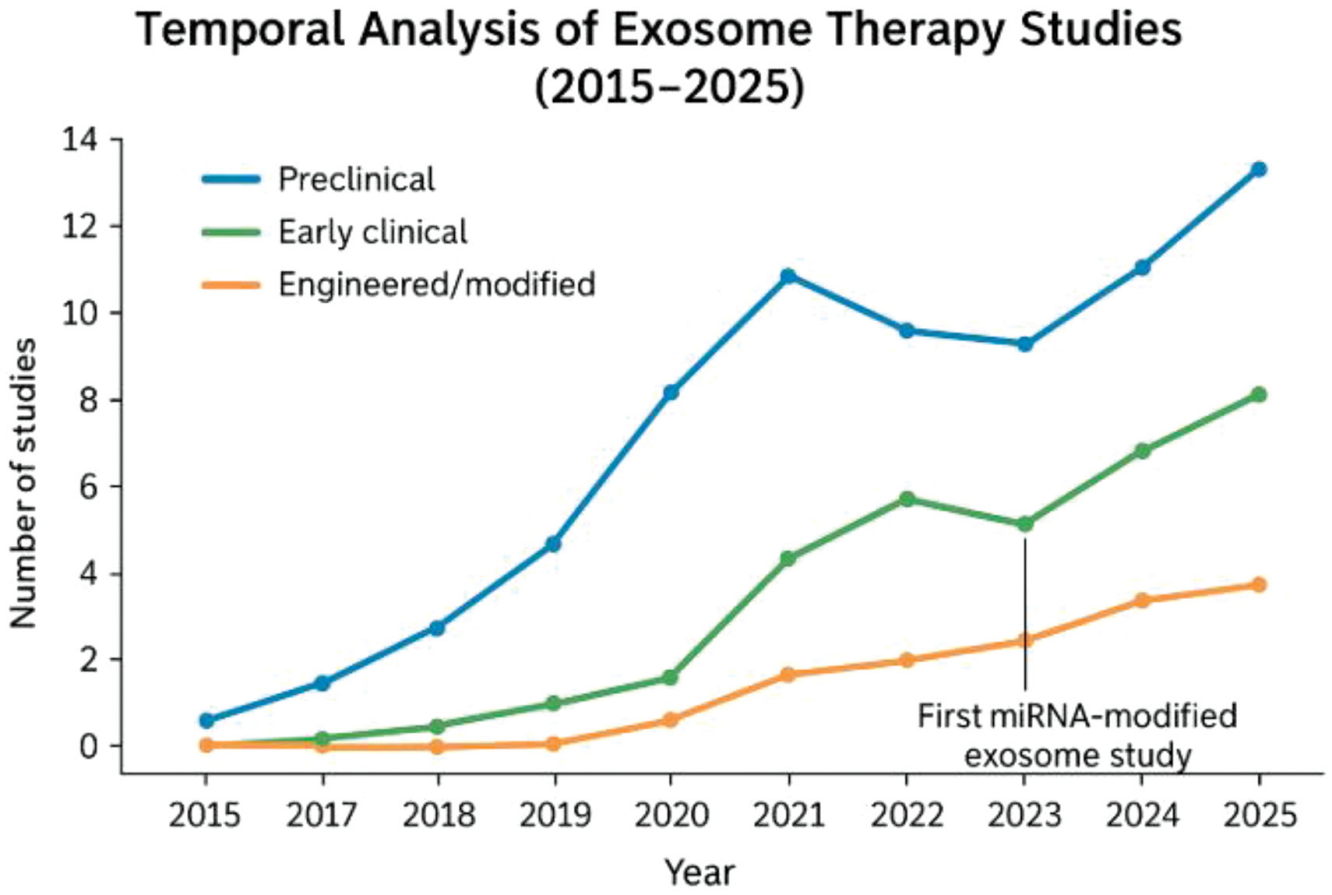
Trends in exosome applications in plastic and reconstructive surgery (2015–2025).

The bibliometric analysis of PubMed-indexed studies (2015–2024) shows that exosome therapy research progressed from early mechanistic animal studies to methodological optimization and, from 2020 onward, early-phase clinical trials—indicating a maturing field advancing toward standardized and translational therapeutic applications, as illustrated in Figure 3. The figure was generated using bibliometric data retrieved from PubMed-indexed records. Only publicly available, peer-reviewed publications were included. No unpublished or original data were used.

This bibliometric analysis of PubMed-indexed publications demonstrates a marked increase in research output, with the annual number of articles rising from approximately 20 in 2015 to over 180 in 2024 (Panel A). Correspondingly, Panel B illustrates a progressive evolution in the nature of published studies across three time periods, characterized by a shift from predominantly preclinical animal models toward increasing proportions of isolation and characterization method papers and early-phase clinical trials, with the search strategy and inclusion criteria detailed in section “8 Evidence synthesis.”

## 9 Safety considerations and regulatory landscape

### 9.1 Regulatory status: FDA and international perspectives

As of 2025, no exosome-based products have received approval from the US Food and Drug Administration (FDA) for therapeutic or cosmetic applications (95). The regulatory status of exosome therapies remains complex and evolving. Many commercial products, particularly those marketed for cosmetic skin rejuvenation, exist within a regulatory gray area, often labeled as “cosmetic” or “wellness” products rather than drugs or biologics (96). This classification allows manufacturers to circumvent rigorous FDA scrutiny, leading to wide availability despite limited safety and efficacy data. Similar trends are observed internationally, with some countries adopting more permissive frameworks for cosmetic-grade exosomes, while others maintain stricter oversight comparable to that for cell and gene therapies (97). The FDA’s 2023 draft guidance explicitly classifies exosome therapeutics as biological products subject to good manufacturing practice (GMP), sterility, potency, and safety requirements (98). However, many commercially available preparations do not meet these standards, posing challenges for clinical translation and patient safety. Regulatory agencies worldwide are still working to develop harmonized frameworks that can keep pace with rapid scientific advances in this field (99). Differences between GMP-certified and non-GMP exosome preparations critically influence clinical reproducibility, particularly regarding sterility assurance, quantification of vesicle potency, and batch-to-batch consistency. Regulatory frameworks such as the FDA (100) and EMA (101) currently classify exosome-based therapies within advanced biologic or cell-derived medicinal product categories, requiring demonstrable manufacturing control, well-defined release criteria, and validated characterization markers. Increasing alignment with GMP standards represents a key step toward ensuring reliable translation of exosome therapies into surgical practice (102).

Globally, regulatory approaches to exosome-based therapeutics differ. The European Medicines Agency (EMA) classifies these products as Advanced Therapy Medicinal Products (ATMPs) (103), requiring Investigational Medicinal Product Dossiers and validated potency assays prior to clinical approval. Japan’s Pharmaceuticals and Medical Devices Agency (PMDA) (104) allows conditional early clinical use under regenerative medicine frameworks if Phase I safety data are available. In contrast, South Korea’s MFDS (101) and China’s NMPA employ hybrid schemes (105), distinguishing exosomes for medical use from those for cosmetic applications, with the latter requiring simplified pre-market notification. This global disparity stems from a fundamental regulatory distinction: products intended for therapeutic claims (e.g., treating a disease like a diabetic ulcer) are universally classified as drugs or biologics, requiring rigorous pre-market approval involving clinical trials and GMP compliance. In contrast, products marketed for cosmetic purposes (e.g., improving skin texture) with no therapeutic claims may fall under less stringent cosmetic regulations in some jurisdictions, focusing primarily on safety and labeling rather than proof of efficacy. This has led to a situation where cosmetic-grade exosome products are commercially available in some markets without the same level of pre-market scrutiny applied to therapeutic exosomes in clinical trials, underscoring the need for greater international harmonization.

### 9.2 Safety concerns

Despite the promising biological effects of exosomes, safety concerns persist. The primary risks include immunogenic reactions, ranging from mild local inflammation to systemic hypersensitivity, though reported incidences remain low in existing clinical studies (106). Additionally, contamination during manufacturing may introduce pathogens, increasing the risk of infection (107). Another potential concern is the pro-tumorigenic capacity of exosomes, as they can carry oncogenic proteins and nucleic acids capable of stimulating malignant cell proliferation or metastasis under certain conditions (108). While preclinical studies highlight this theoretical risk, definitive clinical evidence of exosome-induced malignancy is currently lacking (109). Inflammatory responses, including cytokine release and fibrosis, have also been reported in rare cases following exosome administration (110). At the molecular level, exosome-induced adverse effects appear linked to specific cargo components, such as miR-21 and miR-155, which can activate oncogenic signaling via STAT3 and PI3K/AKT pathways in predisposed tissues. Similarly, exosomes derived from senescent or immortalized cells may transfer DNA damage signals and reactive oxygen species, contributing to local inflammation.

Long-term safety data remain limited, with median follow-up across clinical studies averaging only six months. Continuous pharmacovigilance and mandatory post-marketing surveillance are therefore essential as exosome-based products enter expanded clinical use. Implementing standardized potency and purity testing will mitigate risks of immune activation, infection, and off-target effects. Together, these safeguards will form the basis for responsible clinical integration.

## 10 Proposed roadmap for clinical translation

The clinical integration of exosome therapy in plastic and reconstructive surgery requires a systematic strategy that addresses three principal challenges. Building on the findings of this review, a unified roadmap is proposed to guide translation from preclinical discovery to regulated clinical application, conceptually represented through three interdependent pillars—standardized manufacturing, biomarker-guided patient stratification, and regulatory harmonization. The first priority is the development of standardized manufacturing protocols. Current exosome preparations show considerable variability, with only 23% of studies adhering to International Society for Extracellular Vesicles (ISEV) guidelines (111). Achieving reproducibility requires harmonized particle characterization and molecular profiling of therapeutic cargo such as microRNAs (miRNAs) and growth factors, employing advanced quantification tools like tunable resistive pulse sensing (TRPS) and mass spectrometry. Transitioning from research-grade ultracentrifugation to scalable tangential flow filtration (TFF) systems is essential for good manufacturing practice (GMP) compliance, despite remaining challenges in yield and purity. The second pillar emphasizes biomarker-based patient stratification to enable precision medicine. Early trials demonstrate heterogeneous outcomes, reflecting biological variability among patients. Inflammatory cytokines such as interleukin-6 (IL-6) and tumor necrosis factor-alpha (TNF-α), together with vascular endothelial growth factor (VEGF) expression, may serve as predictive biomarkers for treatment response. For example, patients with diabetes or impaired vascularization may benefit from exosomes enriched in angiogenic factors, whereas individuals at risk of hypertrophic scarring may respond more favorably to vesicles carrying anti-fibrotic miRNAs. The third pillar involves international regulatory harmonization. In the United States, the Food and Drug Administration (FDA) divides oversight between the Center for Biologics Evaluation and Research (CBER) and the Center for Drug Evaluation and Research (CDER); the European Medicines Agency (EMA) classifies exosomes as Advanced Therapy Medicinal Products (ATMPs); and Japan’s Pharmaceuticals and Medical Devices Agency (PMDA) applies more flexible approval pathways. Coordinated global guidelines defining minimal manipulation criteria, product characterization standards, and post-market safety monitoring are needed to streamline translation without compromising patient safety. Collectively, these three pillars form an integrated translational roadmap that replaces the need for a separate schematic figure, providing a cohesive framework for clinical adoption. This strategy directly bridges the gap between promising preclinical evidence and variable clinical outcomes by establishing robust manufacturing standards, enabling biomarker-guided precision approaches, and aligning regulatory oversight. Through this structured pathway, exosome therapy can advance from experimental applications toward reliable, evidence-based integration into reconstructive and regenerative surgical practice.

## 11 Limitations and future directions

The field of exosome therapy in plastic and reconstructive surgery faces several barriers to clinical implementation. Methodological inconsistencies across studies, including variations in exosome sources, isolation techniques, and delivery approaches, create challenges for reproducibility and therapeutic standardization (112). These variations contribute significantly to conflicting clinical outcomes, particularly in applications like scar modulation and wound healing (113). Compounding these issues is the lack of long-term safety and efficacy data, with most studies limited to short-term endpoints of six months or less (114).

To advance the field, researchers must prioritize standardized protocols and rigorous clinical validation. Implementation of good manufacturing practices and adherence to established characterization guidelines will be crucial for ensuring product consistency (115). A shift toward precisely defined therapeutic products with quantified cargo profiles represents a critical step forward from current “black box” approaches (116).

Emerging bioengineering strategies offer promising solutions to current limitations. Targeted modification of vesicle contents and surface engineering could enhance therapeutic precision for specific applications (117). Such innovations may enable more effective treatments for challenging conditions like chronic wounds while addressing issues of biological variability (118). Parallel developments in scalable production methods and improved storage solutions will support broader clinical adoption (119).

Adherence to the Minimal Information for Studies of Extracellular Vesicles (MISEV) guidelines issued by the International Society for Extracellular Vesicles is fundamental for ensuring reproducibility and regulatory alignment in exosome-based therapeutics (120). The MISEV2018 framework requires multiparametric characterization, including particle sizing and quantification (e.g., NTA or TRPS), morphological validation (TEM or equivalent imaging), demonstration of multiple EV-enriched markers (such as CD9, CD63, CD81, TSG101, or Alix), and inclusion of negative markers to exclude non-vesicular contaminants (121, 122). It further emphasizes transparent reporting of purity metrics and standardized dosing definitions to facilitate cross-study comparison and clinical translation. Within the body of literature analyzed in this review, implementation of these criteria remains inconsistent, with frequent reliance on single-marker validation, limited reporting of negative controls, and heterogeneous dose expression formats. Such variability directly affects preparation purity, potency assessment, and comparability of therapeutic outcomes, thereby complicating regulatory classification and approval pathways under advanced biologic frameworks. Systematic adoption of MISEV-compliant characterization standards will therefore be essential to improve methodological transparency, strengthen translational validity, and support harmonized regulatory evaluation of exosome-based interventions in reconstructive surgery.

The evolution of research from 2015 to 2025 demonstrates a clear progression toward clinical translation. This trajectory suggests imminent advances in protocol standardization, completion of pivotal trials, and establishment of regulatory pathways for key indications. Realizing this potential will require continued collaboration across research, clinical, and regulatory domains to bridge the gap between laboratory innovation and patient care.

## 12 Conclusion

Exosome therapy for plastic and reconstructive surgery is poised at a critical translational inflection point, where its future clinical utility hinges not on further biological discovery but on systematically overcoming key bottlenecks: the implementation of universal GMP-compliant manufacturing standards to ensure product consistency and potency, the generation of robust Level I clinical evidence through rigorous trials to establish definitive efficacy and safety, and the development of coherent, harmonized regulatory frameworks to guide safe and effective integration into clinical practice; only through this disciplined, collaborative focus on standardization, evidence, and regulation can exosomes evolve from investigational agents into predictable and powerful tools in regenerative medicine.
